# Effect of Processing Route on Microstructure and Mechanical Properties of an Al-12Si Alloy

**DOI:** 10.3390/ma17194780

**Published:** 2024-09-28

**Authors:** Abdulrahman Alsolami, Adnan Zaman, Fahad Alshabouna, Abdulaziz Kurdi, Ahmed Degnah, Salman Alfihed, Thamer Tabbakh, Animesh Kumar Basak

**Affiliations:** 1 Microelectronics and Semiconductors Institute, King Abdulaziz City for Science and Technology, P.O. Box 6086, Riyadh 11442, Saudi Arabia; aalsolami@kacst.gov.sa (A.A.); salfihed@kacst.gov.sa (S.A.); ttabbakh@kacst.gov.sa (T.T.); 2 Advanced Materials Technology Institute, King Abdulaziz City for Science and Technology, P.O. Box 6086, Riyadh 11442, Saudi Arabia; f.alshabouna@kacst.gov.sa (F.A.); akurdi@kacst.gov.sa (A.K.); adegnah@kacst.gov.sa (A.D.); 3 The Center of Excellence for Advanced Materials and Manufacturing, King Abdulaziz City for Science and Technology, P.O. Box 6086, Riyadh 11442, Saudi Arabia; 4 Adelaide Microscopy, The University of Adelaide, Adelaide, SA 5005, Australia

**Keywords:** powder laser bed fusion, microstructure, micro-pillar compression, Al-12Si (wt. %) alloy

## Abstract

Two different microstructures of an Al-12Si (wt. %) alloy were produced, respectively, via a powder laser bed fusion (P-LBF) additive manufacturing and casting. Compared to casting, additive manufacturing of Al-based alloy requires extra care to minimize oxidation tendency. The role of the microstructure on the mechanical properties of Al-12Si (wt. %) alloy was investigated by in situ compression of the micro-pillars. The microstructure of additively manufactured specimens exhibited a sub-cellular (~700 nm) nature in the presence of melt-pool arrangements and grain boundaries. On the other hand, the microstructure of the cast alloy contains typical needle-like eutectic structures. This striking difference in microstructure had obvious effects on the plastic flow of the materials under compression. The yield and ultimate compressive strength of the additively manufactured alloy were 23.69–27.94 MPa and 75.43–81.21 MPa, respectively. The cast alloy exhibited similar yield strength (31.46 MPa); however, its ultimate compressive strength (34.95 MPa) was only half that of the additively manufactured alloy. The deformation mechanism, as unrevealed by SEM investigation on the surface as well as on the cross-section of the distorted micro-pillars, confirms the presence of ductile and quasi-ductile facture of the matrix and the Si needle, respectively, in the case of the cast alloy. In contrast, the additively manufactured alloy exhibits predominantly ductile fractures.

## 1. Introduction

Different manufacturing processes of a given metallic alloy give rise to different microstructures, and the development of unique microstructure dictates the mechanical characteristics of such alloy. Traditionally, the different microstructure of a given alloy is generated by adding different alloying elements, as well as post-treatments like aging, heat-treating, solution-treating, etc. As a rule of thumb, the general objective of such post-treatments is to allow the precipitation and intermetallic phases to form and distribute throughout the matrix. In such cases, the matrix provides the toughness, and the precipitation/intermetallics act as a load-bearing component, like in the case of metal matrix composites [1,2].

As reported in the literature, additive manufacturing (AM), which is a modern manufacturing practice, can give rise to a unique microstructure of a given alloy, which is not achievable by traditional processing of such alloys. Among various derivatives of the AM process, powder laser bed fusion (P-LBF) [3] is commonly employed for the AM of metallic alloys [4]. In this method, a layer of powder (~50–200 µm thick) is spread out on a support plate, and a laser beam consolidates the powders [5,6] according to the given input parameters. Once the given layer is consolidated, then the next one follows. Thus, this is, in fact, a ‘bottom–up’ approach to building three-dimensional structures by stacking up 2D layers of materials. The whole process is computer-controlled, based on the ‘slicing geometry’ of a given object, and takes place in a confined atmosphere for better control of the process parameters. The final object is a ‘near-net’ shape and, thus, does not require the subsequent post-manufacturing machining process, such as turning, cutting, milling, etc. Thus, this technique offers rapid manufacturing, prototyping, and even onsite production of required components with minimum lead time and inventory. Moreover, material wastage is minimal, as there is no formation of chips, scouts, and cut-offs, which makes the process environmentally friendly [7]. The un-solidified powder from the process can be used as a feedstock for the next cycle and increase the efficiency of the process further [8,9].

Aluminium (Al) alloys are one of the most commonly used structural materials. In the virgin form, aluminum is too soft to use for any structural applications; thus, it has always been alloyed by adding alloying elements or as metal matrix composites (MMC) by incorporating second-phase particles. Al-12Si (wt. %) is a typical lightweight material [10], which is traditionally manufactured by casting. Cast Al-Si alloys commonly find their application in automotive and aerospace industries, where the reduction in specific weights together with high specific strength is desirable. Moreover, the broken parts can easily be recycled and replaced with similar parts at low costs [11,12,13,14]. The high percentage of silicon in this alloy offers additional benefits, such as reducing the melting point, increasing the melt fluidity during processing, and enhancing its resistance against corrosion and wear, with moderate improvement in strength [15]. Research on the traditional way of processing Al-12Si (wt. %) alloy (i.e., casting) was widely reported in the literature [16,17]. However, to exploit the benefits of the additive manufacturing technology, Al-12Si (wt. %) alloy is being prepared by L-PBF additive manufacturing and evaluated mostly in terms of process parameter optimization and microstructural characterization [18,19,20]. Recently, the AM of the Al-12Si (wt. %) alloy has been reported to have the formation of unique microstructures, which are completely different from that of the cast alloy [21,22,23]. Compared to other metallic alloy systems, the AM of Al-12Si alloy is challenging [24,25] due to high thermal reflectivity, as well as conductivity of Al powder bed that rapidly diffuses the heat [26] and high tendency of oxidation. Moreover, the formation of tenacious oxide films causes wetting issues that lead to keyhole pore formations in the consolidated materials [27]. Thus, optimization of the input process parameters is a must, which includes high laser power, slower scan speeds, and close overlapping distances (hatch spacing) [27]. Based on the previous studies, as reported in the literature, most of the work on the L-PBF Al-12Si alloy involves process parameter optimization, together with microstructural investigation. There are only a handful of reports available on their mechanical characteristics, such as tensile properties [18], through conventional macro-scale mechanical testing. With respect to that, in situ compression of micro-pillars was conducted in the present work to investigate the role of microstructure on the mechanical aspects of Al-12Si (wt. %) alloy and one-to-one comparison to that of a bulk alloy of similar compositions. In addition to that, deformation aspects at the micro-/nano-scale were also looked for. To the authors’ best knowledge, there are no such reports available in the literature, which is the novelty of the present work.

The objective of the current research is to examine the role of different microstructures on the mechanical characteristics of such alloys, together with respective deformation behaviors. Toward that, Al-12Si (wt. %) alloy was fabricated via the L-PBF process and cast. Details of microstructure evolution were investigated by using electron microscopy, and the mechanical characteristics were evaluated by in situ compression of micro-pillars. Deformed micro-pillars were also investigated to unravel their deformation mechanism.

## 2. Experimental

The feedstock material for AM was a gas-atomized powder of Al-12Si alloy, which was commercially procured from Valimet Inc., Stockton, CA, USA. The powder particles were in spherical morphology, with a particle-size distribution of 20–60 µm. The selection of the input parameters was based on the energy density (ED) of the system, which is commonly represented as [28,29]

(1) $$ED=\frac{P}{v_{s}ht}\;$$

where *P* is laser power vs. scanning speed; *h* is hatch distance, and *t* is layer thickness. As reported in the literature, the recommended energy densities for the Al-12Si system is about 40–80 J/mm^3^ to avoid overoxidation or lack of fusion. In view of that, 55 J/mm^3^ energy density was maintained during AM, with the help of the following process parameters [10,23]: 320 W laser power; 0.1 mm hatch spacing; 0.1 mm layer thickness; and 120 mm/min scan speed. The AM was performed in an inert atmosphere of Ar in an SLM 250 HL AM unit (SLM Solutions Group AG, Lübeck, Germany). The laser source was Nd:YAG, with a 400 W capacity. To ease sample removal, the support plate was pre-heated to 200 °C, together with a 45° rotation of scan direction between consecutive layers, to avoid thermal stress build-up. The resulting specimen was a 10 mm cube. After that, the cubic specimen was heated at 240 °C for stress relief [30].

The Archimedes’ principle was applied to measure the density of the specimens. After that, phases of the materials were studied by an X-ray diffractometer (XRD) using CuKα monochromatic radiation (New D8 advance, Bruker, Karlsruhe, Germany). Then, the cubes were cut in the middle with the help of a water-cooled saw, followed by hot mounting in Struers automatic hot-mounting press (CitoPress-15, Struers, Ballerup, Denmark) in a conductive resin powder. After that, the resin blocks were subjected to grinding and metallographic polishing using Struers’ automatic metallographic polisher (Struers, Denmark). Different grades of diamond slurry were employed during polishing, with the final polishing on colloidal silica. The microstructural examination of the specimens was obtained by the use of a scanning electron microscope (FESEM, Hitachi SU 7000, Tokyo, Japan) equipped with X-ray dispersive spectroscopy (EDS). Cross-section analyses of the distorted micro-pillars were performed by a focused ion beam (FIB) technique (Zeiss cross-beam 350, Zeiss, Aalen, Germany). In situ compression of the micro-pillars was conducted inside the SEM chamber with the help of the Hysitron PI 88 nanoindentation (Bruker, Tucson, AZ, USA) system mounted with a flat diamond punch. The loading rate of compression was selected as 3 nm/s, which translates into 10^−3^ s^−1^ strain rate. After compression, the cross-section of the distorted micro-pillars was also carried out by the FIB technique (Helios Nanolab 600, Thermofisher Scientific, Waltham, CA, USA).

## 3. Results and Discussion

The density of a given alloy indicates how well the specimens were fabricated in the absence of porosity and voids. In that respect, the density of the produced specimens was calculated according to Archimedes’ method and reported in Table 1. According to the literature [10], the theoretical density of the Al-12Si is 2.65 g/cc. Against that, L-PBF fabricated Al-12Si alloy attained 97.3% of the theoretical density. Thus, there is room for further improvement in that respect by manipulating input parameters. In contrast, cast alloy attains 99.6% of theoretical density.

**Figure 1:**
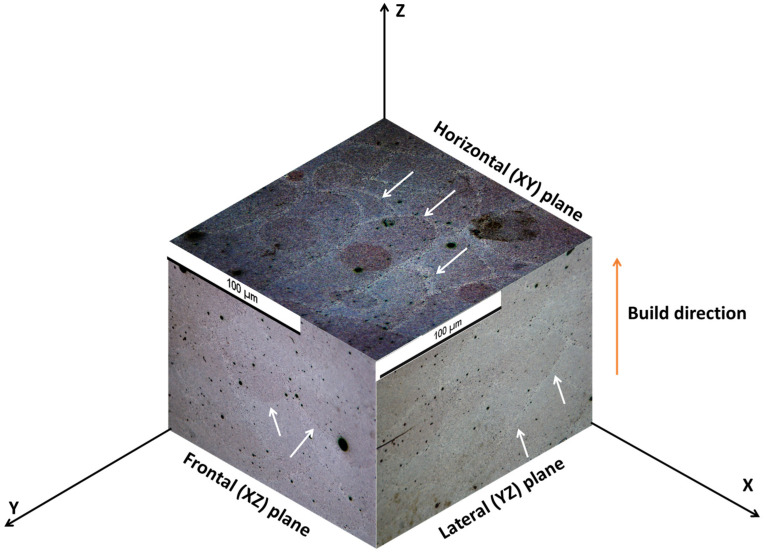
Three-dimensional ‘isometric’ view of the L-PBF Al-12Si (wt. %) alloy by optical microscopy.

According to the literature, AM-fabricated specimens may show anisotropy in microstructure [31,32]. Thus, to have an overall view of that, AM-processed specimens were investigated from three sides with respect to the build direction. The direction vertical to the build direction is referred to as the horizontal (XY) plane, and the planes parallel to the build direction are denoted as frontal (XZ) and lateral (YZ) planes, as depicted in Figure 1.

### 3.1. Microstructure of the Alloys

A three-dimensional view of the additively manufactured specimen is depicted in Figure 1 by incorporating optical images obtained from different directions. The path of the laser tracks appears as a typical ‘fish scale’-type appearance in the microstructure [33], as shown by the white arrows in Figure 1. In addition to that, the boundaries of successive laser tracks seem like melt-pool-type appearances [34,35], which are 100’s of microns in width.

**Figure 2:**
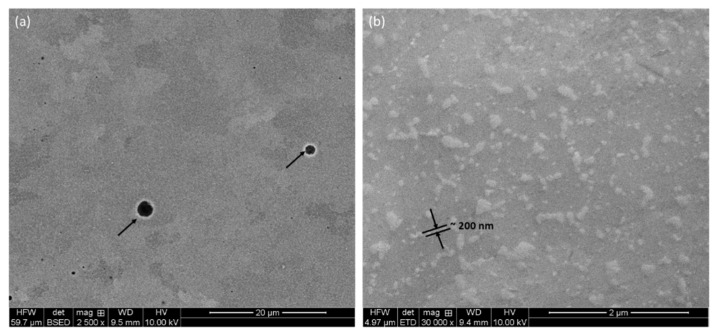
Backscattered electron (BSE) micrographs of the P-LBF-processed Al-12Si alloy at different magnifications on horizontal plane: (**a**) 2500× and (**b**) 30,000×.

**Figure 3:**
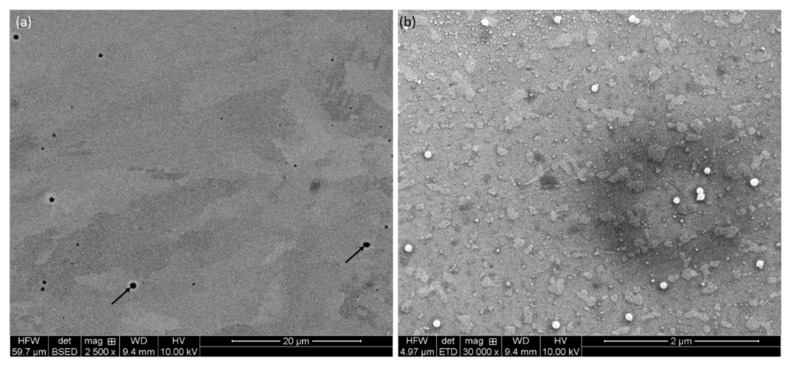

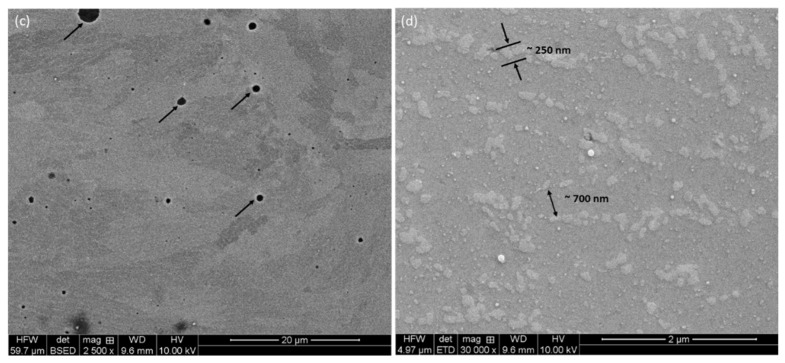
(**a**,**c**) Backscattered electron (BSE) and (**b**,**d**) secondary electron (SE) micrographs of the P-LBF-processed Al-12Si alloy at different magnifications.

Details on microstructure on different planes were further examined by backscattered electron (BSE) micrographs, as shown in Figure 2 and Figure 3, at different magnifications. Irrespective of the directions, metallurgical pores are present throughout, as shown by black arrows in Figure 2a and Figure 3a,c. These pores appear due to gas-bubble entrapment, where the cooling rate is high, and the gas bubbles do not have the time to escape the melt [36,37]. These pores preferentially reside at the interface of melt-pool boundaries, which is the last region of the melt to be solidified [25], as stated in the literature; however, in the present investigation, it seems that the pores are thoroughly distributed in the structure. As suggested in the literature, these can be limited either by manipulating input parameters or by post-treatment of the components [21,38,39].

As evident from the high-magnification micrographs (Figure 2b and Figure 3b,d), Si is finely discrete in Al-matrix. The matrix itself is made of grain boundaries, as well as melt-pool junctions. Inside the melt-pool junctions, the grains are equiaxed in appearance, as this region was subjected to multiple heat cycles due to the overlapping of the laser tracks. Inside the melt-pool region, it gives rise to cellular/sub-cellular type microstructures [38] in the range of about 700 nm (Figure 3d). This development of the microstructures was only possible due to the presence of superheating of the melt, followed by quick solidification. Due to superheating, Si solubility in the melt increases. As the Al matrix starts to solidify in a dendritic nature, the solute in the melt is pushed into the spaces between the dendritic arms. As the Si concentration keeps rising in those confined areas (space between dendritic arms), Si is rejected from the melt and takes place in those areas [38]. As reported by Li et al. [21], the core of the melt pool may reach a temperature up to about 1712.15 K. However, the limited time of interaction, together with minimum melt volume, facilitates inhomogeneous microstructural formations in melt-pool boundaries [21]. As reported by Prashanth et al. [38], the cell size ranges from 500 to 1000 nm, and the thickness of the cell boundaries is about 200 nm. A similar trend was also noticed in the present case (Figure 2 and Figure 3), where the cell size was about 700 nm with 200–250 nm of cell boundary thickness.

**Figure 4:**
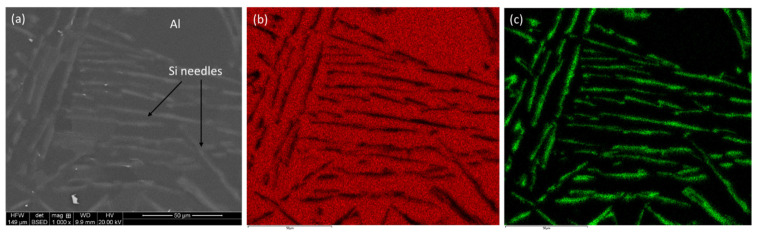
SEM micrograph (**a**) and elemental mapping of Al (**b**) and Si (**c**) in a cast Al-12Si (wt. %) alloy.

In contrast, the microstructure of the cast alloy is completely different. Instead of a finely dispersed Si in the Al matrix, large Si needles are distributed, which are about 100’ microns long, with a thickness of about 1–2 µm. This is a typical eutectic structure of a cast Al-12Si (wt. %) alloy, which was further verified by elemental mapping (Figure 4b and Figure 4c, respectively, for Al and Si). As confirmed by elemental mapping, the Si needles are randomly distributed in the matrix.

### 3.2. Phase Characterization

**Figure 5:**
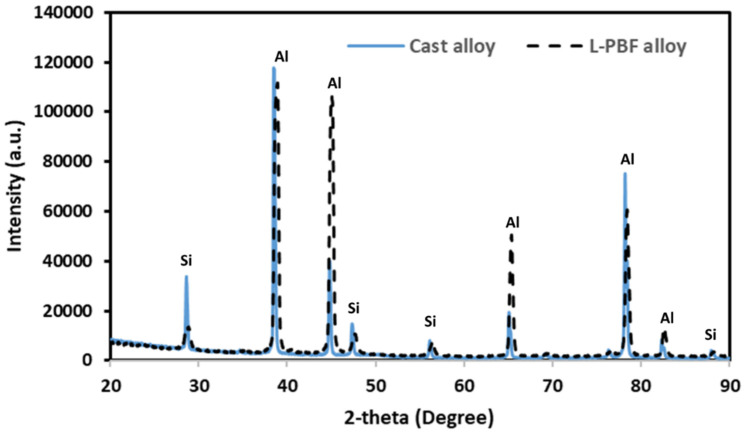
X-ray diffraction spectra on L-PBF and cast Al-12Si (wt. %) alloy.

The different phases that were present in the microstructure of the alloys were investigated by XRD. The XRD spectrum of L-PBF and cast Al-12Si (wt. %) are shown in Figure 5. Based on the XRD spectrum, it can be concluded that there were no phase changes or development of any phases as a result of different processing conditions. The only phases present are (a) α-Al and (b) Si phase. The reason for such simplicity is that Al and Si do not form any compound or intermetallic phases, according to the Al-Si phase diagram [40]. This particular alloy composition is a near-eutectic in nature composition, where the Al and Si solidify in an alternative fashion. The change in Si morphology (fine disperse vs. needle phase) is a time-dependent phenomenon, where the fine dispersion is attributed to a higher solidification rate. This aspect does not necessarily impact the rise of any particular phase, as confirmed by the XRD spectra.

### 3.3. Mechanical Properties of the Alloys

#### 3.3.1. Micro-Pillar Fabrication

**Figure 6:**
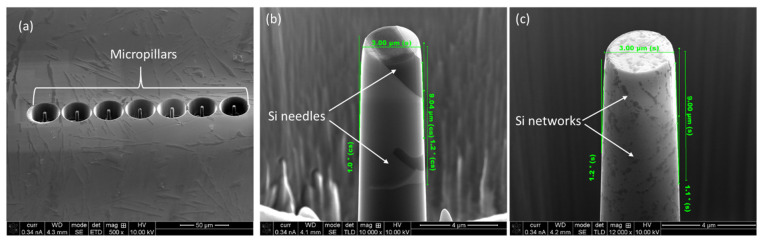
A series of fabricated micro-pillars on cast Al-12Si (wt. %) alloy (**a**), together with an enlarged 45° view of micro-pillar on (**b**) cast and (**c**) L-FBF-processed alloy (horizontal plane), indicating the dimensions of the micro-pillar and existence of Si needles/networkers.

A focused ion beam (FIB) technique was employed to make the micro-pillars on the polished samples. Initially, a 6.5 nA current at 30 kV was used to mill away large volumes of the material (ϕ 30 μm), with progressively a lower current with final polishing at 0.46 nA current at 30 kV to obtain a smooth surface finish. The dimensions of the micro-pillars were 3 μm in diameter, with a length of 9 μm to keep an aspect ratio of 1:3 to avoid buckling during compression [41]. As shown in Figure 6a, seven individual micro-pillars on a given material and plane were made and subsequently compressed for data reproducibility. The micro-pillars were sightly tapered (<2°), as shown in Figure 6b,c, which was unavoidable due to the ion beam-material interaction [42]. As shown in Figure 6b, in the case of cast Al-12Si alloy, a given micro-pillar contains several Si needles (marked with white arrows). On the other hand, micro-pillars in the L-PBF-processed alloy contain numerous Si networks. Thus, the micro-pillars are representative of the given materials.

#### 3.3.2. Compression of Micro-Pillars

**Figure 7:**
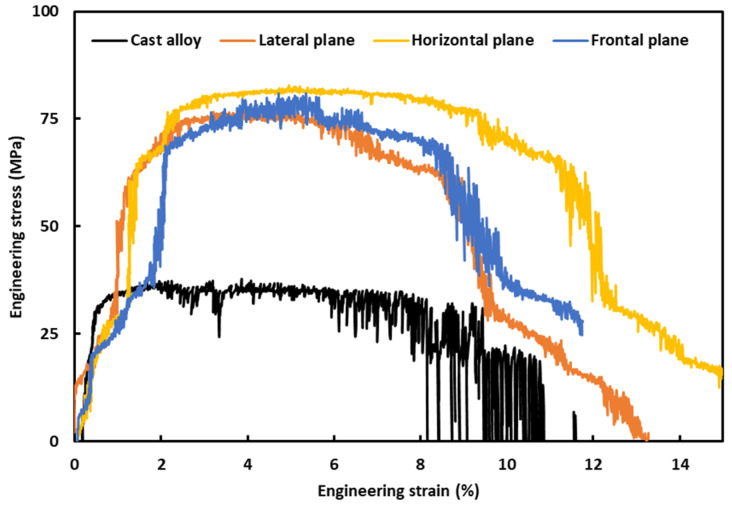
Engineering stress–strain graphs on L-PBF and cast Al-12Si (wt. %).

The compression of the micro-pillars was conducted inside the SEM in a precise manner, and the computer controller system recorded the corresponding load and displacement. The load-displacement data were later used to plot the stress–strain curves, according to the calculations reported in the previous communication [43], which takes into consideration the ‘Sneddon effect’ [44]. Figure 7 exhibits the average engineering stress–strain curves on L-PBF Al-12Si (wt. %) on different planes, as well as on cast alloy as a benchmark material. Though several micro-pillars were compressed for a given material, only single stress–strain curves of the given material were included in Figure 7 for neatness and ease of comparison.

At least seven micro-pillars were compressed for a given material and plane; however, single stress–strain curves of the given material and plane were included in Figure 7 for neatness and ease of comparison. Having said that, data obtained from all the micro-pillar compressions for a given material and plane were taken into consideration, and average values of the mechanical properties with corresponding standard deviation are reported in Table 1. As can be seen from Figure 7, for any given strain level, the stress of the cast alloy is much lower than that of the L-PBF-processed alloy. The evolution of the stress–strain curves is typical of that of ductile metallic materials [45], where the stress rises substantially with strain at the beginning of loading before reaching a plateau. In the plateau region, the material holds the stress level, being continuously strained. At that point, the applied load is beyond the accommodation capability of the material, and thus, a drop in the stress level takes place as a strain continues until a complete collapse (fracture) of the material. There are numerous sudden stress drops in the course of compression, which can be ascribed to the start and subsequent dissemination of the slip/shear planes. This is more prominent in the case of L-PBF alloy, as there is a break within 2% of strain. The reason behind that will be more evident in the next section, where the post-deformation SEM images of the micro-pillars were presented. These stress–strain curves were further analyzed to retrieve some key mechanical aspects, as given in Table 1.

As can be deduced from Table 1, the yield strength of the cast alloy is marginally higher (31.46 MPa) than L-PBF-processed alloy (23.69–27.94 MPa). In the case of the Young’s modulus, L-PBF alloy possesses about half of the Young’s modulus of the cast alloy. However, this scenario is completely opposite in the case of ultimate compressive strength, where the L-PBF-processed alloy possesses 75.43–81.21 MPa, compared to 34.95 MPa of the cast alloy. Thus, the ultimate compressive strength of the L-PBF-processed alloy is about two times higher than that of the cast alloy. This implies that though the yield strength of the L-PBF-processed alloy is similar to that of cast alloy, the toughness of the L-PBF-processed alloy is much higher, as indicated by the higher ultimate compressive strength. The underlying reason for such behavior is related to the development and dissemination of the slip/shear planes, together with the accommodation of the applied loads by the given materials, as discussed in the next section. The data reported in the present investigation are also backed up by the findings of other researchers, where a similar material was subjected to traditional tensile testing. For example, Misra et al. [41] reported up to 22 MPa of yield stress and up to 25 MPa of ultimate tensile stress of the cast alloy. However, in the case of L-PBF-processed alloy, a wide range of values were reported, such as 20–260 MPa for yield strength and 75–450 MPa [18,42,43,44] for ultimate tensile stress, as different researchers have utilized different feedstock powders, and different input parameters, as resources permit.

In the case of L-PBF alloy, different planes exhibit similar evolution of the stress–strain curves, together with key mechanical properties (Table 1), which lie within standard deviation (experimental errors). Thus, the anisotropy is not prominent in the present case.

#### 3.3.3. SEM of Compressed Micro-Pillars

**Figure 8:**
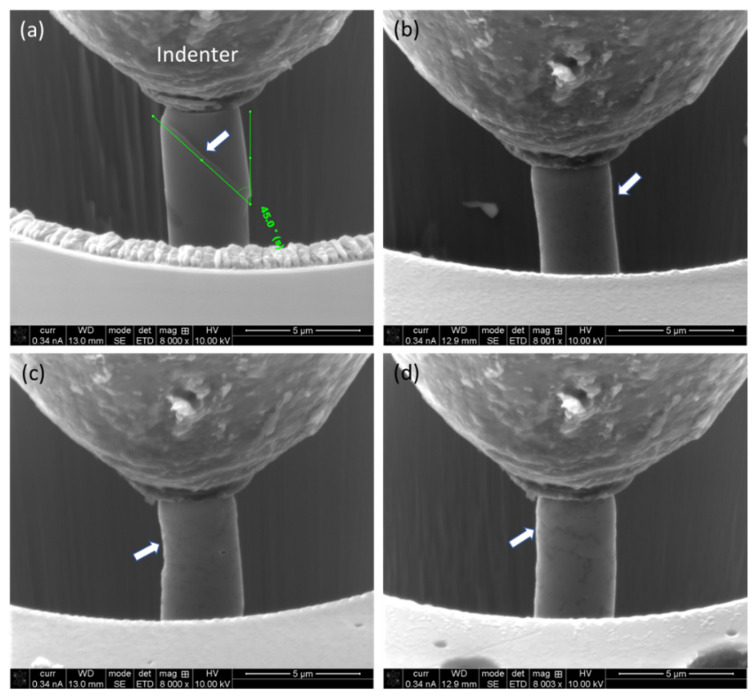
Stage of the micro-pillars in course of loading at 5% intervals on cast alloy (**a**) and different planes on L-PBF-processed alloy: (**b**) horizontal plane; (**c**) lateral plane; and (**d**) frontal plane. The white arrows indicate the locations of slip/shear planes.

SEM micrographs were recorded both in the course of compression and after completion of the compression. Figure 8 shows the stages of the deformation of the micro-pillars at about a 2% strain interval. As evident from Figure 8, the micro-pillar on the cast alloy exhibits a clear formation of sharp slip/shear planes, which is about 45° to that of the loading direction. However, for L-PBF alloy, irrespective of a given plane, there is no such formation of a slip/shear plane. Instead of that, the matrix (Al) ‘squeezed’ out through the Si network, with the formation of shallow shear planes in the vicinity of the Si network, as marked with white arrows in Figure 8b–d. This deformation mechanism was responsible for the relatively lower ultimate compressive strength of the cast alloy to that of the L-PBF alloy. Also, it is worth noting that the Si appears as a needle (and plates in three dimensions) in the cast alloy, which facilitates the movement of the slip/shear planes. This phenomenon is somewhat similar to that reported in the case of high-entropy alloy [46,47] and segregation of alloying elements in grain boundaries in additively manufactured Inconel alloys [48,49]. This physical evidence sheds light on the inherent deformation behavior of these two differently processed materials, which will be discussed in the next section.

**Figure 9:**
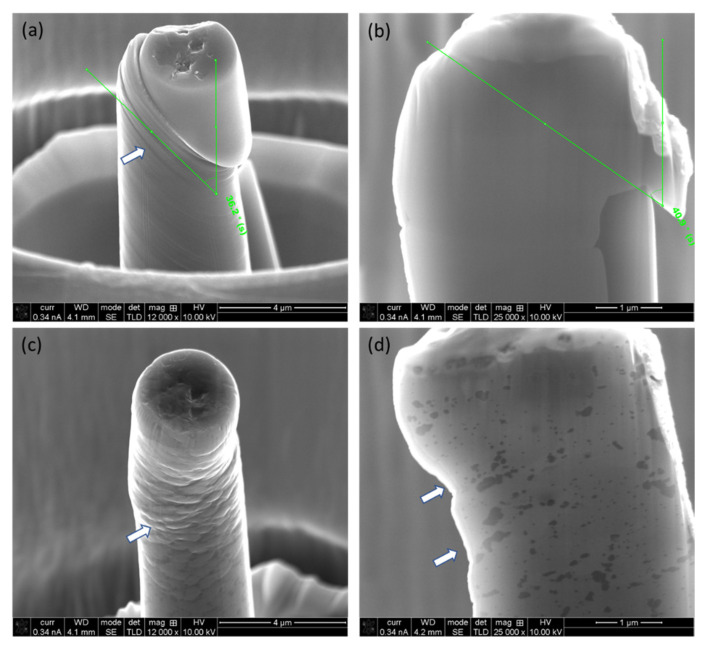
SEM micrograph of surface morphology (**a,c**) and cross-section (**b,d**) of distorted micro-pillars on cast alloy (**a,b**) and L-PBF alloy (**c,d**) on horizontal plane. The white arrows indicate the locations of slip/shear.

The distorted micro-pillars were analyzed further by SEM after the completion of loading on both surface morphological and cross-sectional points of view, as shown in Figure 9.

As evident from the surface (Figure 9a,c) and cross-section (Figure 9b,d) view of the micro-pillar on the cast alloy, the slip/shear plane is prominent and very sharp, which lies at about 40°, with respect to the loading direction. Also, there are numerous slip/shear planes, and deformation takes place by the displacement of the ‘disk’ of materials. In contrast, the L-PBF-processed alloy (on a horizontal plane, Figure 9c,d) exhibits the ‘squeezing out’ of materials instead of forming shape slip planes, as evident from the cross-sectional view (Figure 9d).

### 3.4. Deformation Mechanism under Compression

As reported in Section 3.3.2, the yield strength of the L-PBF-processed alloy is similar to that of cast alloy. However, the ultimate compressive stress (i.e., the toughness of the L-PBF alloy) is much higher. As indicated in Section 3.3.2 and later confirmed by SEM micrographs of the compressed micro-pillars in Section 3.3.3, this is related to the deformation phenomena of the micro-pillars during compression. As can be understood from Figure 8a and Figure 9a,b, in the case of cast alloy, the deformation mechanism involved the formation of high-angle (~40–45°) slip/shear planes, which are mostly confined in the interphase of the Si needle, surrounding the matrix material (Al), and very sharp in nature. This gives rise to the quasi-ductile behavior of the fracture surface. On the other hand, for L-PBF alloy, the slip/shear planes are not very well-defined and imply fully ductile modes of fracture. It seems that instead of forming sharp slip/shear planes, the matrix (Al) material is squeezed out through the network of Si cell/grain boundaries (Figure 2 and Figure 3) and exhibits a pure ductile mode of fracture. This also explains the obtained mechanical properties of materials, as reported in Table 1. It takes a similar level of strength (i.e., yield strength) for both materials for the initial deformation. In the case of the cast alloy, once the deformation initiates, there is nothing to hold it back, as the interface of dissimilar elements (Si and Al) favors the propagation of the slip/shear planes, which gives rise to sharp, high-angle slip planes. On the other hand, in the cast of L-PBF-processed alloy, there are no such interphases of Si needles with an Al matrix. Instead, the Si is finely discrete in a metal (Al) matrix, forming a kind of network-like structure and holding it tight. As the hardness of the Si is much higher (13 GPa [50]) than the matrix Al (160–350 MPa [51]), instead of breaking the Si network, the Al is squeezed out through the network. This gives rise to a higher ultimate compressive strength material. However, as the loading continues, at some point, the Si networks give up about >8% of strain, and the stress drops substantially and proceeds toward the final separation in the form of fracture.

## 4. Conclusions

The microstructure and mechanical aspects of L-PBF Al-12Si (wt. %) alloy were studied and evaluated against cast alloy of similar composition. It was obvious that the microstructure of L-PBF alloy, which was made of cellular structure, was exclusive, together with melt-pool boundaries. This difference in microstructure impacted the mechanical aspects of the alloy, as uncovered by in situ micro-pillar compression. The L-PBF alloy possesses about 23.69–27.94 MPa of yield strength and 75.43–81.21 MPa of ultimate compressive strength. Though the cast alloy possesses a similar level of yield strength (31.46 MPa), a much lower ultimate compressive strength (34.95 MPa) is about half that of additively manufactured alloy. The comparatively lower ultimate compressive strength of the cast alloy was associated with the deformation mechanism, which was substantially different from that of the L-PBF-processed alloy. The cast alloy exhibited ductile and quasi-ductile fractures of the matrix and the Si needle, respectively, in contrast with the predominantly ductile fracture of additively manufactured alloy, where the Si network provided the ultimate toughness.

## Figures and Tables

**Table 1:** Key aspects of the presently investigated alloys.

Properties	P-LBF-Processed Al-12Si (wt. %) Alloy			Cast Al-12Si (wt. %) Alloy
	Lateral (YZ) Plane	Frontal (XZ) Plane	Horizontal (XY) Plane	
**Density (gm/cc)**	2.60			2.63
Yield strength (σ_y_), MPa	23.69 ± 3.35	24.61 ± 2.36	27.94 ± 4.87	31.46 ± 3.25
Ultimate compressive strength (σ_UTS_), MPa	75.43 ± 8.39	78.68 ± 8.98	81.21 ± 7.38	34.95 ± 6.12
Young’s modulus (GPa)	36.89 ± 4.35	31.89 ± 3.95	37.41 ± 4.12	80.64 ± 5.65

## Data Availability

The raw data supporting the conclusions of this article will be made available by the authors on request.
